# Energy-dependent modulation of nucleus accumbens output via K-ATP channel activity

**DOI:** 10.1038/s41386-026-02429-8

**Published:** 2026-04-30

**Authors:** Simone Astori, Olivia Zanoletti, Jocelyn Grosse, Carmen Sandi

**Affiliations:** 1 Laboratory of Behavioral Genetics, Brain Mind Institute, School of Life Sciences, Ecole Polytechnique Fédérale de Lausanne, Lausanne, Switzerland; 2 Synapsy Center for Neuroscience and Mental Health Research, School of Life Sciences, Ecole Polytechnique Fédérale de Lausanne, Lausanne, Switzerland

**Keywords:** Cellular neuroscience, Motivation

## Abstract

Mitochondria are central to neuronal bioenergetics, supporting the high metabolic demands required for synaptic signaling and network activity. Yet how neurons adapt their activity to rapid fluctuations in energy supply—and how such adaptations shape behavior—remains poorly understood. We previously showed that acute pharmacological manipulation of mitochondrial complex activity in the nucleus accumbens (NAc) affects motivated behaviors, which led us to hypothesize that medium spiny neurons (MSNs) can rapidly adjust their output in response to bioenergetic levels. To test this hypothesis, we examined how acute mitochondrial inhibition alters MSN function using mouse brain slices. Inhibition of mitochondrial complex I with the selective inhibitor rotenone reduced MSN intrinsic excitability, an effect that was counteracted by intracellular ATP replenishment. We next asked whether ATP-sensitive potassium (K-ATP) channels, canonical regulators of membrane excitability under metabolic stress, contribute to these responses. Histological analyses revealed specific expression of Kir6.2 subunits in both D1- and D2-MSNs, as compared to non-MSNs, and electrophysiological recordings showed that K-ATP channel activation blockade prevented rotenone-induced reductions in MSN excitability. In behavioral assays, complex I inhibition impaired effort-related performance, an effect that was rescued by K-ATP channel blockade. These findings identify K-ATP channels in MSNs as key mediators that sense acute changes in neuronal energy state and translate them into rapid adjustments in NAc excitability and behavior.

## Introduction

Mitochondria are central to neuronal function, acting as key regulators of energy production, calcium homeostasis, and oxidative stress [1–4]. Dysregulation of mitochondrial dynamics and bioenergetics has been implicated in the pathophysiology of neuropsychiatric and neurodegenerative disorders [5–10]. Across these disorders, a core and often disabling dimension is disrupted motivated behavior, implicating mesolimbic circuits as a central locus [11, 12].

Within the mesolimbic system, the nucleus accumbens (NAc) has been shown to be a key site where mitochondrial activity critically shapes motivated behavior and vulnerability to mood disorders [13–15]. Medium spiny neurons (MSNs), the predominant neuronal subtype in the NAc, rely heavily on intact mitochondrial function to maintain dendritic complexity, excitatory inputs, and intrinsic excitability [16–21]. Emerging work shows that alterations in mitochondrial function in the NAc can result from constitutive differences in mitochondrial function, as observed in rodent models of anxiety [16, 17, 22], or from chronic manipulations such as deletion of mitochondrial proteins [19, 23]. However, NAc neurons are also acutely sensitive to transient mitochondrial perturbations, as shown by pharmacological manipulations in the NAc that transiently inhibit mitochondrial complexes or boost respiration, which bidirectionally alter the outcome of a social competition [16]. These findings suggest that rapid shifts in mitochondrial energy output directly engage mechanisms of excitability, allowing the NAc to adapt its functional output in real time according to cellular energy availability. Elucidating these mechanisms is crucial not only for understanding how the NAc calibrates behavior in relation to energy states [11, 24], but also for revealing fundamental mechanisms by which neurons rapidly adjust to fluctuations in metabolic resources.

Here, we investigated whether and how acute inhibition of mitochondrial function impacts NAc MSN excitability, with a particular focus on energy-related mechanisms. To this end, we employed ex vivo electrophysiology to assess how acute inhibition of mitochondrial complex I by low-dose rotenone—widely used experimental inhibitor that blocks electron transfer at complex I and thereby impairs ATP synthesis—modulates the excitability of NAc MSNs. We further hypothesized that increasing intracellular ATP or blocking ATP-sensitive potassium (K-ATP) channels—well-established energy sensors regulated by ADP/ATP ratios [25]—would mitigate the effects of mitochondrial inhibition. K-ATP channels are composed of four transmembrane-spanning potassium channel subunits (Kir6.1 and Kir6.2), each associated with a regulatory sulphonylurea receptor (SUR1 or SUR2) [26]. When the ADP/ATP ratio increases, as it occurs during metabolic stress or increased neuronal activity, K-ATP channels open, leading to membrane hyperpolarization and decreased neuronal excitability. To address whether this mechanism is engaged in accumbal MSNs, we examined the expression of K-ATP channel subtypes and tested their activation directly or downstream of complex I inhibition. Finally, we investigated how complex I inhibition in vivo influenced coping strategies in a behavioral paradigm involving the exertion of effort to face inescapable stress and whether pharmacological blockade of K-ATP channels could reverse this outcome. Our findings demonstrate that K-ATP channels act as rapid molecular sensors linking cellular energy states to MSN excitability and behavioral output. Through this mechanism, they enable the NAc to dynamically effort-based behavior in response to fluctuations in metabolic demand.

## Materials and methods

### Animals

Animal care and experimental procedures were conducted in accordance with the Swiss Federal Guidelines for Animal Experimentation and were approved by the Cantonal Veterinary Office Committee for Animal Experimentation (Vaud, Switzerland, license no. VD3454 and VD3778). C57BL/6 J and B6.Cg-Tg(Drd1a-tdTomato)6Calak/J (Drd1a-tdTomato) mice of either sex were used. Mice were maintained on a 12:12 h light–dark cycle with free access to water and standard chow.

### Ex vivo electrophysiology

Mice of 5-12 weeks of either sex were anesthetized with isoflurane and decapitated. The brain was quickly removed and placed in oxygenated (95% O_2_/5% CO_2_) ice-cold modified artificial cerebrospinal fluid (aCSF), containing (in mM): 105 sucrose, 65 NaCl, 25 NaHCO_3_, 2.5 KCl, 1.25 NaH_2_PO_4_, 7 MgCl_2_, 0.5 CaCl_2_, 25 glucose, 1.7 L(+)-ascorbic acid. Coronal slices (250 µm thick) containing the ventral striatum were cut using a vibrating tissue slicer (Campden Instruments) and incubated in standard aCSF containing (in mM): 130 NaCl, 25 NaHCO_3_, 2.5 KCl, 1.25 NaH_2_PO_4_, 1.2 MgCl_2_, 2 CaCl_2_, 18 glucose, 1.7 L(+)-ascorbic acid, and complemented with 2 Na-pyruvate and 3 myo-inositol. The incubation temperature was ~35°C during the first hour. In the recording chamber, slices were superfused with oxygenated standard aCSF at nearly physiological temperature (30-32°C). Patch-clamp recordings of intrinsic excitability were conducted from neurons in the NAc in the whole-cell configuration using borosilicate pipettes (3-4 MΩ) filled with a ‘low ATP’ solution containing (in mM): 135 mM KGluconate, 10 mM KCl, 10 mM HEPES, 0.2 mM EGTA, 1.5 mM Mg-ATP, 0.2 mM Na-GTP. In a subset of experiments, an ‘ATP-regenerating’ solution was used, containing (in mM): 130 KGluconate, 10 KCl, 10 HEPES, 0.2 EGTA, 10 phosphocreatine, 4 Mg-ATP, 0.2 Na-GTP (290-300 mOsm, pH 7.25-7.3). To elicit neuronal firing, cells were held at -60 mV in current clamp configuration with direct current injections, and depolarization was provided by 2-s long current steps of increasing magnitude. Automatic bridge compensation was applied. For evoked inhibitory postsynaptic currents (eIPSCs), the aCFS was supplemented with 0.01 mM DNQX and 0.1 mM D,L-APV, and NAc shell neurons were patched in the voltage-clamp mode using pipettes filled with (in mM): 100 KCl, 40 KGluconate, 10 HEPES, 10 phosphocreatine, 0.4 EGTA, 2 Mg-ATP, 0.2 Na-GTP, 2.5 QX-314-Cl^-^ (290-300 mOsm, pH 7.25-7.3). For evoked excitatory postsynaptic currents (eEPSCs), the aCFS was supplemented with 0.1 mM picrotoxin, and NAc shell neurons were patched in the voltage-clamp mode using pipettes filled with (in mM): 120 CsGluconate, 10 CsCl, 10 HEPES, 10 phosphocreatine, 5 EGTA, 4 Mg-ATP, 0.2 Na-GTP (290-300 mOsm, pH 7.25-7.3). Synaptic currents were evoked by electric stimulation via a bipolar tungsten electrode placed near the recorded cells. Rotenone was dissolved in DMSO and bath-applied for >20 min before patch-clamp recordings. NN414 and Glibenclamide were dissolved in DMSO and bath-applied for >10 min before patch-clamp recordings. DMSO had a final concentration 0.01% in the aCSF and was also included in control recordings. Electrophysiological data were acquired through a Digidata1550A digitizer. Signals were amplified through a Multiclamp700B amplifier (Molecular Devices), sampled at 20 kHz and filtered at 10 kHz using Clampex10 (Molecular Devices). Data were analysed using Clampfit10 (Molecular Devices).

### In situ hybridization RNAscope

For in situ hybridization (RNAscope), C57BL/6 J mice were decapitated, and the extracted brains were snap-frozen in isopentane at –30 °C and kept at –80 °C until further processing. Fluorescent in situ hybridization (FISH) was performed using a RNAscope Fluorescent Multiplex 2.0 assay and RNAscope Probes for *Drd1* (dopamine receptor 1, Mm-Drd1a-C3, no. 406491-C3), *Drd2* (dopamine receptor D2, Mm-Drd2-C2, no. 406501-C2), and *Kir6.1* (inwardly rectifying K^+^ channel 6.1, Mm-Kcnj8, no. 411391) or *Kir6.2* (inwardly rectifying K^+^ channel 6.2, Mm-Kcnj11-O1, no. 431451), as per the manufacturer’s instructions (Advanced Cell Diagnostics). Briefly, cryosections (18 μm) containing the NAc were prepared and mounted on SuperFrost Plus slides. Sections were fixed and pretreated according to the RNAscope guide for snap-frozen tissue. After pretreatment, sections were hybridized with FISH probes using a HybEZ Hybridization System. After several amplification sets, the sections were counterstained with 4,6-diamidino-2-phenylindole (DAPI; Sigma, D9542) and mounted using Prolong Gold. Confocal images were acquired on an LSM 700 confocal microscope (Carl Zeiss) with a 20X/0.8 NA air objective (Bioimaging and Optics Platform, BIOP, EPFL). Cell detection and mRNA quantification were performed using QuPath0.4.4 for cell segmentation and probe detection. Briefly, single nuclei were automatically detected based on the DAPI staining, and cell segmentation was performed with a cell expansion of 5 μm around the nucleus. Cells were classified as *Drd1*+ or *Drd2*+ if the signal intensity in the corresponding channel exceeded a predefined threshold. The expression of Kir6.1 or Kir6.2 was quantified as the average number of probe-specific fluorescent dots per cell.

### Hematoxylin-eosin (HE) staining

HE staining was performed as described previously [16]. Briefly, brain slices (30 μm) were mounted on a slide and dehydrated using increasing steps of EtOH. Sections were incubated with hematoxylin for 5 min, gently rinsed in tap water for 10 min, and stained in eosin for 2 min. Sections were rinsed in distilled water, dehydrated, and mounted. Images were captured using a brightfield slide scanner (Olympus Slide Scanner VS.120-L100).

### Intracerebral cannulation surgery

Male mice aged 9–10 weeks subjected to pharmacological experiments were implanted with bilateral guide cannulas aimed at the NAc. Before surgery, mice received an injection of analgesic (buprenorphine, 0.1 mg/kg) and were anesthetized by isoflurane inhalation (induction 4% isoflurane and maintenance 2.5% isoflurane in O_2_) and placed in a stereotaxic apparatus (David Kopf Instruments). Small holes were drilled through the skull for bilateral placement of stainless steel 26-gauge guide cannula with pedestal (PlasticsOne, #C235GS-5-2.0) fitted with a removable dummy cannula. Coordinates were based on the atlas of Paxinos and Franklin [27] and were taken from bregma (in millimeters): A.P., +1.5; M.L., ±0.75; D.V., −3.50. Cannulas were fixed to the skull with one anchoring screw and dental acrylic (DuraLay Reliance, #2244). Mice were single-housed after surgery, treated with paracetamol (200–300 mg/kg, Dafalgan) via the drinking water for 7 days after the surgery, and allowed to recover for at least 10 days. For intracerebral infusions, the dummy was removed, and a 33-gauge injector (Plastics One, #C235IS-5) that extended 1 mm from the tip of the cannula was inserted aiming at the NAc. Drug infusions were performed in freely moving mice in a housing cage. Animals were habituated to the experimenter through repeated handling during the week preceding the behavioral assays, which minimized stress during the procedure. Behavioral experiments were performed 20 min after drug administration. Animals were randomly assigned to their respective treatment. All drugs were bilaterally infused in a total volume of 0.3 μl during 1 min of constant flow (0.3 μl/min). The injector remained in place for one additional minute after infusion to allow proper diffusion. Rotenone and glibenclamide were dissolved at a concentration of 0.21 mM [16] and 0.1 mM, respectively, in DMSO. Mice in the vehicle group were bilaterally infused with DMSO. After behavioral experiments, animals were euthanized by decapitation, and correct cannula placement was routinely verified with Cresyl violet histology. Images were captured using a bright-field slide scanner (Olympus VS120-L100). Three mice that were not successfully targeted into the NAc were excluded from analysis.

### Forced swim test

Forced swim test (FST) was performed to assess coping behavior. The FST apparatus used was a cylinder of transparent plastic containing water at 23 ± 1°C. The cylinder was half-filled, in order to prevent the mouse from touching the bottom of the cylinder with its paws or tail. The animal was placed in the water for 6 min. The %immobility (i.e., absence of active escaping behavior) was scored by two experimenters blind to treatment, and the results were averaged.

### Open field

Animals were placed in a rectangular arena (50 cm × 50 cm) that they were free to explore for 10 min. Lighting was maintained at 8–10 lux on the center of the arena. Video tracking of the animal’s location was performed by a camera fixed above the arena and automatically scored using the Ethovision tracking system (Ethovision 11.0 XT, Noldus, Information Technology). Open field and FST were conducted in separate groups of animals.

### Statistics

Statistical analyses were performed using GraphPad Prism 10.6. Data distribution was assessed for normality using the Shapiro–Wilk test. For normally distributed data, comparisons were performed using unpaired Student’s *t* tests or one- or two-way ANOVA, as appropriate. When significant effects were detected in ANOVA, appropriate post hoc multiple comparisons tests were applied, as specified in the figure legends. For data not meeting normality assumptions, non-parametric tests (Mann–Whitney or Kruskal–Wallis tests) were used.

Data are presented as mean ± SEM. Individual data points represent single observations. The number of cells (n) and the number of animals (N) are indicated in the figure legends. Statistical significance was set at α < 0.05.

## Results

### Mitochondrial complex I inhibition reduces MSN intrinsic excitability

**Fig. 1:**
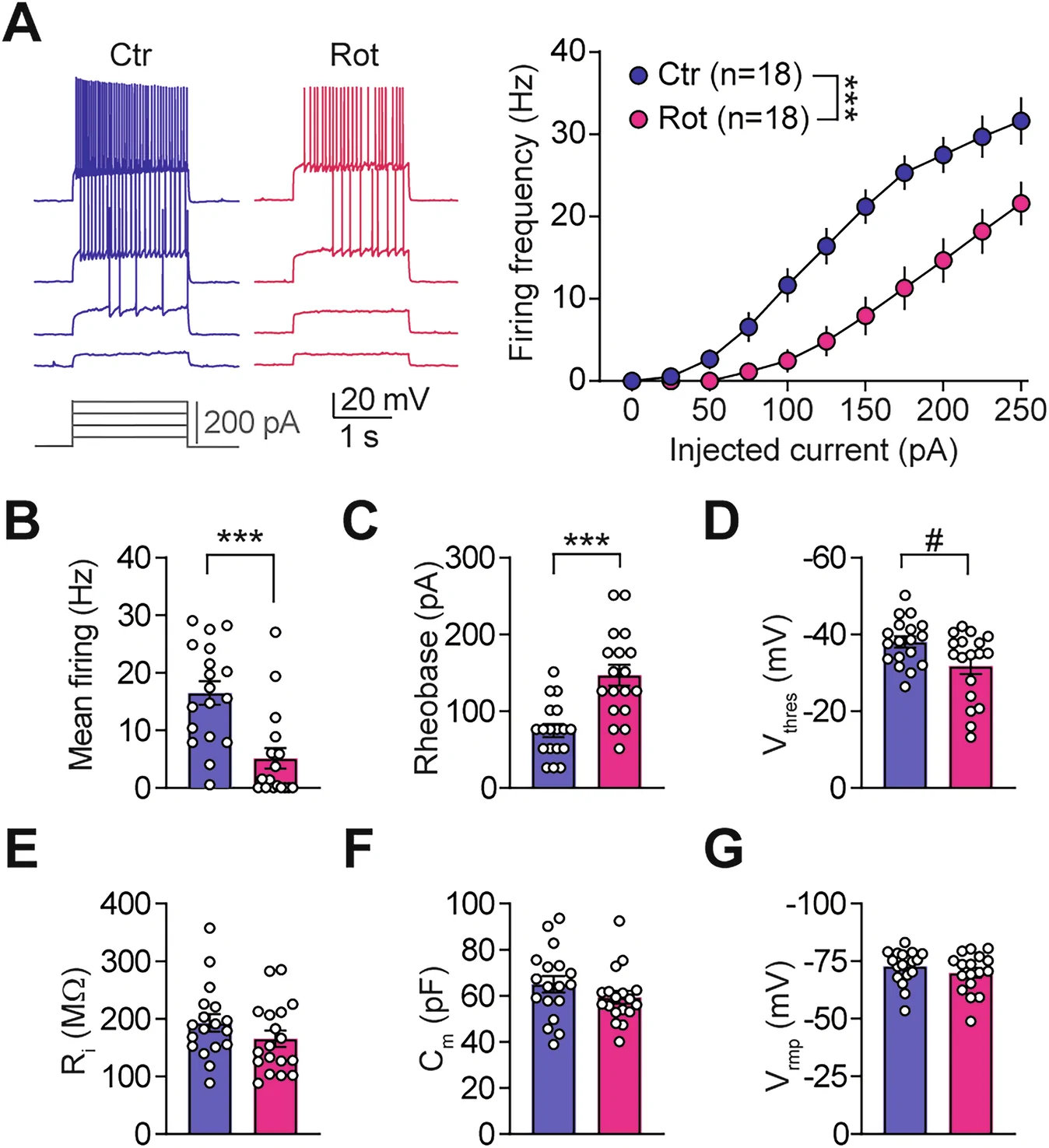
Mitochondrial complex I inhibition reduces the intrinsic excitability of NAc MSNs. **A** Left, examples of voltage responses elicited in NAc MSNs upon somatic current depolarizations (protocol at the bottom). MSNs were preincubated with vehicle (Ctr) or 100 nM rotenone (Rot). Right, average firing frequency vs. current relationships (F-I curves) for Ctr and Rot cells, showing a significant reduction in firing of MSNs treated with rotenone (two-way RM ANOVA; main treatment effect, *F*_(1, 34)_ = 16.93, *p* = 0.0006). **B** Complex I inhibition reduced MSN mean firing frequency (two-tailed Mann-Whitney test, *U* = 45, *p* < 0.0001), quantified as the average firing rate evoked by 100–150 pA depolarizing current injections. **C** Complex I inhibition increased the rheobase of MSNs (two-tailed unpaired *t* test, *t*_34_ = 4.548, *p* < 0.0001), and tended to depolarize the threshold potential (**D**, two-tailed Mann-Whitney test, *U* = 45, *p* = 0.055). **E**–**G** Rotenone did not affect passive membrane properties such as input resistance (R_i_, two-tailed unpaired *t* test, *t*_34_ = 1.326, *p* = 0.19), cell capacitance (C_m_, two-tailed unpaired *t* test, *t*_34_ = 1.241, *p* = 0.22), and resting membrane potential (V_rmp_, two-tailed unpaired *t* test, *t*_33_ = 1.055, *p* = 0.30). Data are represented as mean ± SEM, with datapoints corresponding to single cells; *n* = 18 cells per groups from *N* = 4 male and *N* = 3 female mice. Symbols indicate statistical difference between groups: #*p* < 0.1; ***p < 0.001.

To address how acute in mitochondrial inhibition alters neuronal function, we started by investigating intrinsic excitability of the main neuronal population in the NAc, the MSNs, through patch-clamp recordings in brain slices from mice of either sex. Experimental conditions were chosen in order to minimize the impact of cell dialysis on cytoplasmic ATP levels: recordings were conducted within 5 min from membrane disruption, with an intracellular solution containing a low concentration of ATP (1.5 mM), and data were analyzed by comparing drug- with vehicle-treated cells. Preincubation of the slices (20–60 min) with the mitochondrial complex I inhibitor rotenone at 100 nM—a concentration known not to induce neurotoxicity [16]—significantly decreased MSN firing elicited by depolarizing current injections, compared to control cells preincubated with the vehicle (Fig. 1A). The reduction in mean firing frequency (quantified as the average firing rate evoked by 100–150 pA depolarizing current injections; Fig. 1B) was accompanied by an increase in the rheobase (i.e., the minimal current required to elicit firing, Fig. 1C), and a tendency towards more depolarized threshold potential (Fig. 1D). Importantly, exposure to 100 nM rotenone did not alter cell passive properties, such as membrane resistance, capacitance and resting potential (Fig. 1E–G), which suggests that the observed effects on excitability are not due to cellular toxicity, unlike the significant impacts typically described for higher concentrations of rotenone (e.g., 1 µM [28]). The main effects of rotenone on excitability parameters remained significant when data from male and female mice were analyzed separately, indicating consistent effects across sexes (Fig. S1A–F). Notably, exposure to 100 nM rotenone did not affect the excitability of NAc cells that were classified as putative cholinergic interneurons (Fig. S1G–I) based on the presence of a hyperpolarization-mediated sag in their voltage response [29], suggesting a cell-specific sensitivity of NAc neurons to complex I inhibition.

### Cell-type-dependent impact of complex I inhibition

To investigate potential cell-type differences in the effects of mitochondrial inhibition, we performed experiments in mice from the *Drd1a*-tdTomato line, which enables identification of dopamine receptor 1–expressing MSNs (D1-MSNs) via red fluorescence.

**Fig. 2:**
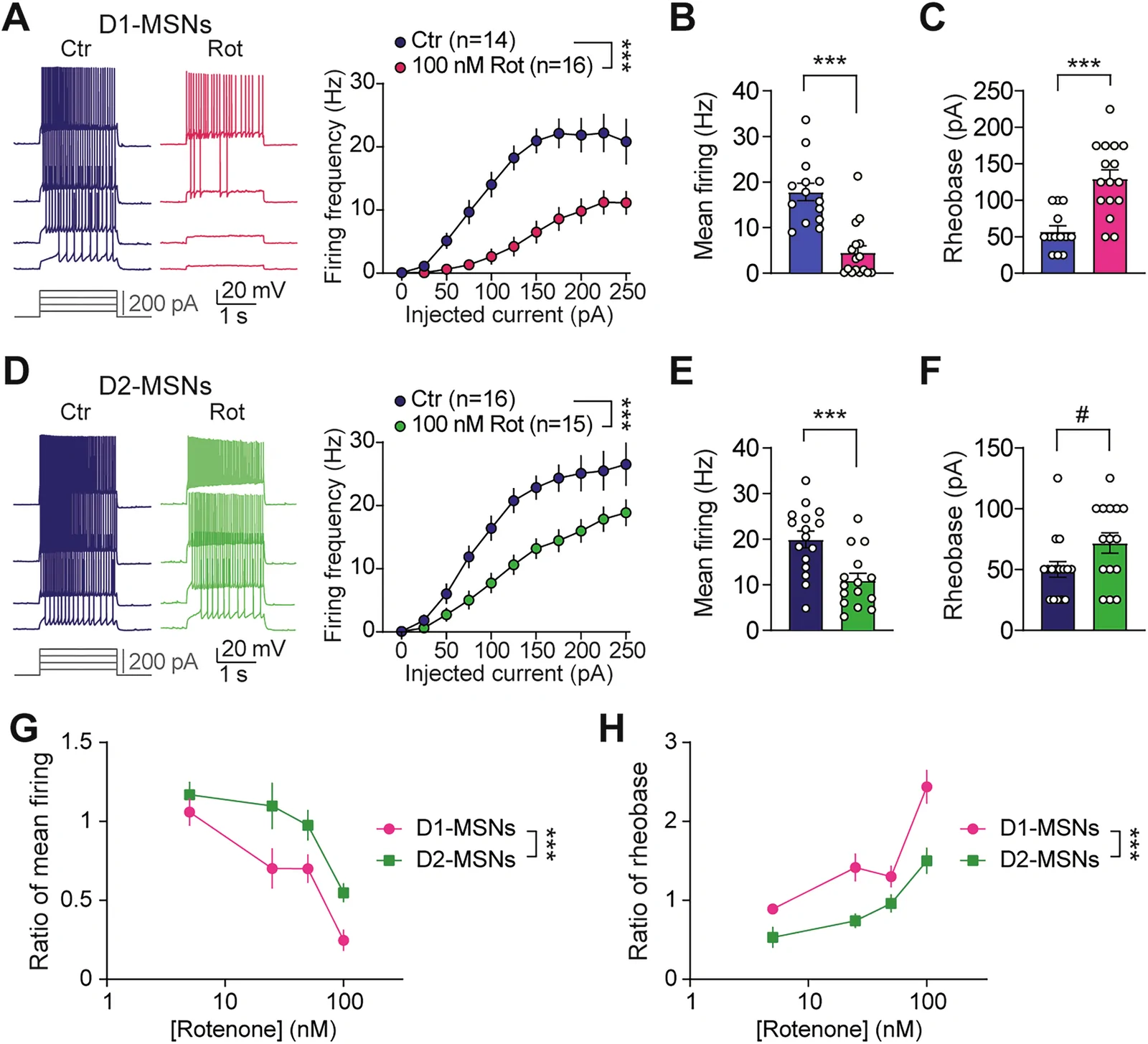
D1-MSNs are more sensitive to mitochondrial I inhibition than D2-MSNs. **A** Left, Examples of voltage responses elicited in NAc D1-MSNs (red fluorescent cells in slices from the Drd1a-tdTomato mouse line), upon somatic current depolarizations (protocol at the bottom). D1-MSNs were preincubated with vehicle (Ctr) or 100 nM rotenone (Rot). Right, F-I curves for Ctr and Rot cells, showing a significant reduction in firing of D1-MSNs treated with 100 nM rotenone (mixed-effects model; main treatment effect, *F*_(1, 27)_ = 35.28, *p* < 0.0001); *n* = 14, 16 cells from *N *= 5 male and *N* = 2 female mice. **B** Mean firing of D1-MSNs was significantly decreased by 100 nM rotenone (two-tailed unpaired t test, t_29_ = 3.7, *p* = 0.22). **C** Rheobase of D1-MSNs was affected by 100 nM rotenone (two-tailed Mann–Whitney test, *U* = 22, *p* < 0.0001). **D** Left, Examples of voltage responses elicited in putative NAc D2-MSNs (MSNs lacking red fluorescence in slices from the Drd1a-tdTomato mouse line), upon somatic current depolarizations (protocol at the bottom). D2-MSNs were preincubated with vehicle (Ctr) or 100 nM rotenone (Rot). Right, F-I curves for Ctr and Rot cells, showing a significant reduction in firing of D2-MSNs treated with 100 nM rotenone (mixed-effects model; main treatment effect, *F*_(1, 30)_ = 14.11, *p* = 0.007); *n* = 16, 15 cells from *N* = 6 male and *N* = 2 female mice. **E** Mean firing of D2-MSNs was significantly decreased by 100 nM rotenone (two-tailed Mann-Whitney test, *U* = 18, *p* < 0.0001). **F** Rheobase of D2-MSNs showed a tendency to increase with 100 nM rotenone (two-tailed Mann-Whitney test, *U* = 73, *p* = 0.056). **G** Dose–response relationships for mean firing frequency, constructed by normalizing the firing frequency values (mean of 100-150 pA steps) of cells exposed to rotenone to the average firing frequency of the respective control conditions. D1-MSNs showed greater sensitivity to rotenone compared to D2-MSNs (two-way ANOVA; main treatment effect, *F*_(3, 73)_ = 20.26, *p* < 0.0001). D1-MSN cell number for Ctr, Rot: 5 nM: *n* = 10, 5 from *N* = 3 female mice; 25 nM, *n* = 10, 11 from *N* = 3 male and *N* = 2 female mice; 50 nM: *n* = 13, 13 from *N* = 1 male and *N* = 2 female mice; 100 nM, *n* = 14, 15 from *N* = 5 male and *N* = 2 female mice. D2-MSN cell number for Ctr, Rot: 5 nM: *n* = 7, 5 from *N* = 2 female mice; 25 nM, *n* = 8, 7 from *N* = 2 male and *N* = 1 female mice; 50 nM: *n* = 11, 11 from *N* = 1 male and *N* = 2 female mice; 100 nM, *n* = 16, 14 from *N* = 6 male and *N* = 1 female mice. **H** Dose–response relationships for rheobase, constructed by normalizing the rheobase values of cells exposed to rotenone to the average rheobase value the respective control conditions. D1-MSNs showed greater sensitivity to rotenone compared to D2-MSNs (two-way ANOVA; main treatment effect, *F*_(3, 73)_ = 19.31, *p* < 0.0001). Data are represented as mean ± SEM, with datapoints corresponding to single cells. Symbols indicate statistical difference between groups: #*p* < 0.1; ***p < 0.001.

In D1-MSNs, 100 nM rotenone markedly reduced firing frequency by ~75% and increased rheobase, indicating a decrease in intrinsic excitability (Fig. 2A–C). In putative D2-MSNs (i.e., Tomato-negative MSNs), 100 nM rotenone reduced firing frequency by ~50% and tended to increase rheobase (Fig. 2D–F). Dose–response relationships were constructed using multiple concentrations of rotenone, with mean firing frequency and rheobase values normalized to their respective control conditions. This analysis showed a greater reduction in excitability in D1-MSNs compared to D2-MSNs in the range of concentrations tested, indicating higher sensitivity of D1-MSNs to mitochondrial inhibition (Fig. 2G–H).

These findings indicate that D1- and D2-MSNs differ in the processes by which mitochondrial complex I inhibition translates into reduced excitability, suggesting cell-type-specific ionic mechanisms downstream of mitochondrial impairment.

### ATP supplementation counteracts the effect of complex I inhibition

**Fig. 3:**
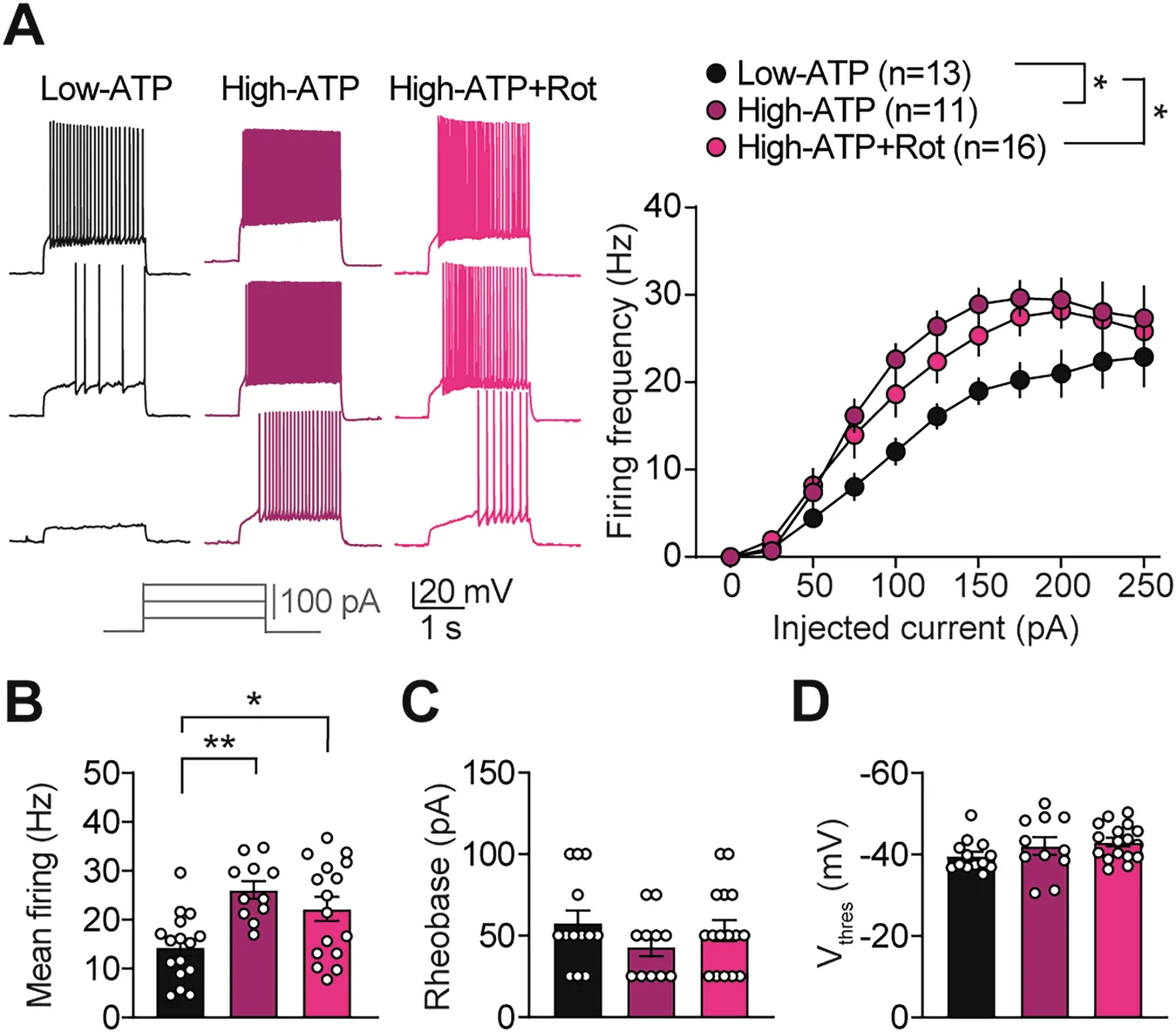
ATP replenishment prevents the effects of rotenone. **A** Left, examples of voltage responses elicited in D1-MSNs upon somatic current depolarizations (protocol at the bottom). D1-MSNs were patch-clamped with an intracellular solution containing 1.5 mM ATP (Low-ATP), or 4 mM ATP and 10 mM phosphocreatine (High-ATP). Right, F-I curves showing that High-ATP significantly increased firing compared to Low-ATP, and prevented the effect of 100 nM rotenone (two-way RM ANOVA; main treatment effect, *F*_(2, 37)_ = 4.759, *p* = 0.0145; Holm-Šídák’s *post hoc* test, Low-ATP vs. High-ATP, *p* = 0.0187; High-ATP vs. High-ATP+Rot, *p* = 0.4471; Low-ATP vs. High-ATP+Rot, *p* = 0.0454). **B** Mean firing frequency was increased in the High-ATP group, and unaltered when treating High-ATP D1-MSNs with 100 nM rotenone (one-way ANOVA, F_(2, 40)_ = 7.881, *p* = 0.0013; Holm-Šídák’s multiple comparisons test, Low-ATP vs. High-ATP, *p* = 0.0016; High-ATP vs. High-ATP+Rot, *p* = 0.22; Low-ATP vs. High-ATP+Rot, *p* = 0.015). Datapoints in the bar graph are mean firing frequencies elicited by the 100-150 pA depolarizing steps. **C**–**D** Rheobase and threshold potential were not different across conditions (rheobase: Kruskal-Wallis test, *K-W* = 1.77, *p* = 0.413; V_thres_: Kruskal-Wallis test, *K-W* = 4.01, *p* = 0.135). Data are represented as mean ± SEM, with datapoints corresponding to single cells; *n* = 13, 11, 16 cells from *N* = 5, 2, 2 male and *N* = 2, 1, 1 female mice. Symbols indicate statistical difference between groups: *p < 0.05; **p < 0.01.

Given that mitochondrial complex I activity is critical for sustaining oxidative phosphorylation and, hence, ATP production, we reasoned that supplementing ATP via the patch pipette into NAc MSNs should mitigate the effects of rotenone on intrinsic excitability. Compared with recordings with a low-ATP intracellular solution (‘Low-ATP’), as in the previous experiments, D1-MSNs displayed higher firing rates when patch-clamped with an ATP-regenerating intracellular solution (’High-ATP’, see Materials and Methods) (Fig. 3). Notably, under this ATP-regenerating condition, the effect of rotenone was largely attenuated (’High-ATP+Rot’) (Fig. 3A–D), indicating that elevating intracellular ATP levels prevents the reduction in excitability induced by complex I inhibition. These results suggested the involvement of ATP-sensitive potassium (K-ATP) channels, which are gated by changes in ADP/ATP ratio, leading to dampening of neuronal excitability when ATP decreases [25].

### NAc MSN express Kir6.2-containing K-ATP channels

**Fig. 4:**
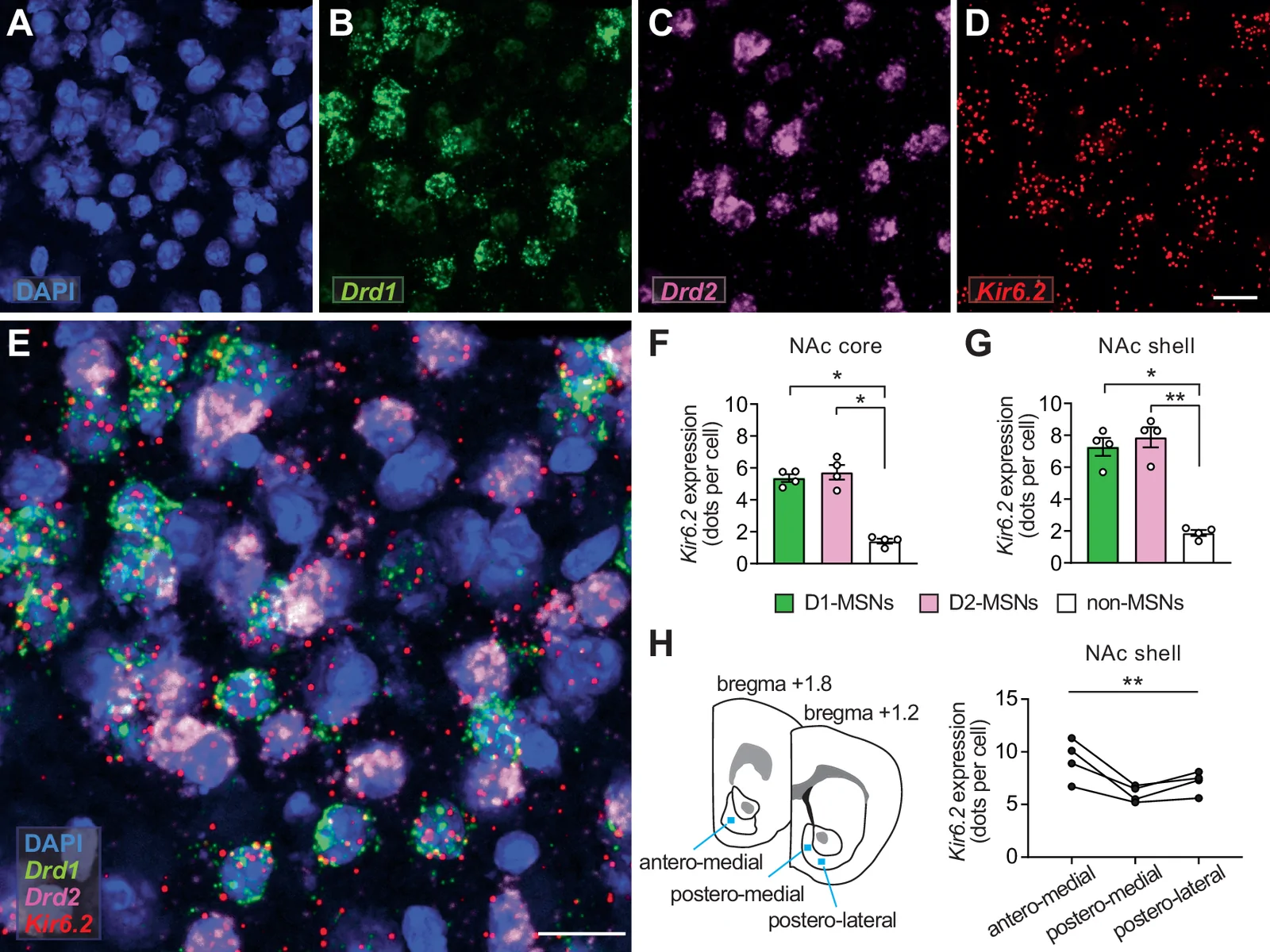
NAc MSNs express Kir6.2-containing K-ATP channels. **A–E** Example images of DAPI staining and in situ mRNA hybridization for *Drd1*, *Drd2*, and *Kir6.2* mRNA labeling in the NAc, with merged channels in **E**, showing that both D1-MSNs and D2-MSNs express Kir6.2-containing K-ATP channels. Scale bars are 20 µm. **F**,** G** Quantification of *Kir6.2* mRNA in the NAc subdivisions, indicating low expression of the Kir6.2 subunit in cells classified as non-MSNs (core: Kruskal-Wallis test, *K-W* = 8, *p* = 0.0099; Dunn’s post hoc test, D1-MSNs vs. D2-MSNs, *p* = 0.694; D1-MSNs vs. non-MSNs, *p* = 0.031; D2-MSNs vs. non-MSNs, *p* = 0.0106; shell: Kruskal-Wallis test, *K-W* = 8, *p* = 0.048; Dunn’s *post hoc* test, D1-MSNs vs. D2-MSNs, *p* = 0.433; D1-MSNs vs. non-MSNs, *p* = 0.0499; D2-MSNs vs. non-MSNs, *p* = 0.006). **H** Quantification of *Kir6.2* mRNA across the antero-posterior axis indicated higher expression of the Kir6.2 subunit in the anteromedial portion of the NAc (RM ANOVA, *F*_(2, 6)_ = 13.41, *p* = 0.0061). Data are represented as mean ± SEM, with datapoints corresponding to single animals (*N* = 4 male mice). Symbols indicate statistical difference between groups: *p < 0.05, **p < 0.01.

To address this possibility, we first characterized the expression of K-ATP channel subunits in NAc MSNs using in situ hybridization for *Kir6* subunits in *Drd1*-positive (D1-MSNs) and *Drd2*-positive (D2-MSNs). The *Kir6.1* subunit was not detected in MSNs, appearing instead in other scattered cells (Fig. S2), consistent with its known expression in cholinergic interneurons [30]. By contrast, the *Kir6.2* subunit was highly expressed in both MSN subtypes across the NAc core and shell, but was virtually absent in non-MSN cell types (Fig. 4A–G). *Kir6.2* expression was particularly enriched in anterior sectors of the NAc (Fig. 4H).

**Fig. 5:**
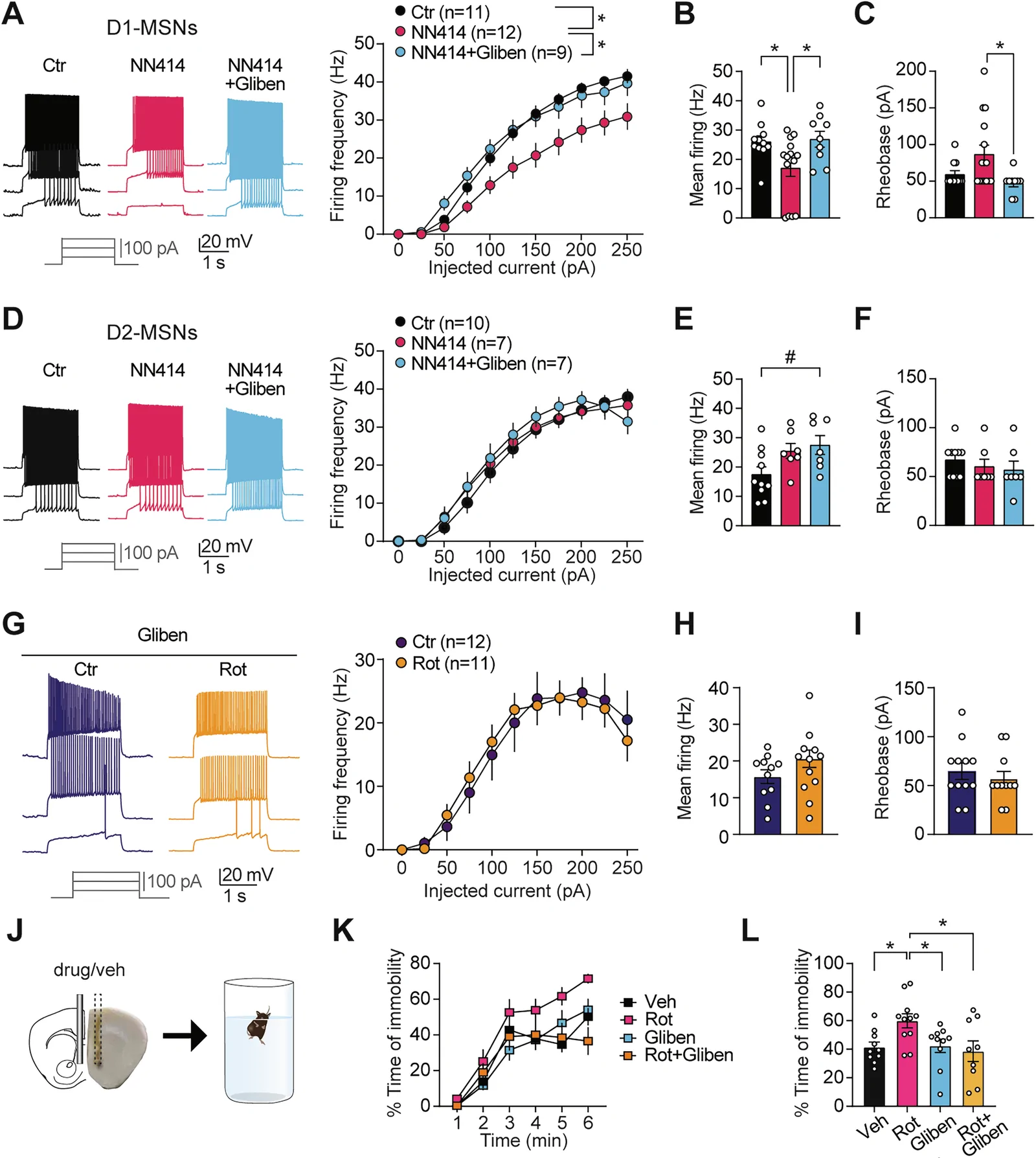
Activation of K-ATP channel activation mimics, whereas their blockade counteracts, the effect of rotenone on MSN intrinsic excitability. **A** Left, representative voltage responses recorded from D1-MSNs in response to somatic depolarizing current injections (protocol shown below). Cells were patch-clamped with an intracellular solution containing 1.5 mM ATP under control conditions (Ctr), in the presence of the K-ATP channel activator NN414 (20 µM), or with NN414 in combination with the K-ATP channel blocker glibenclamide (Gliben, 20 µM). Right, F–I curves showing that K-ATP channel activation decreased D1-MSN firing, an effect that was reversed by K-ATP channel blockade (two-way RM ANOVA; main treatment effect, *F*_(2, 32)_ = 4.896, *p* = 0.014; Holm-Šídák’s multiple comparisons test, Ctr vs. NN414, *p* = 0.0357; NN414 vs. NN414+Gliben, *p* = 0.0357; Ctr vs. NN414+Gliben, *p* = 0.95); *n* = 11, 12, 9 cells from *N* = 3 male and *N* = 1 female mice. **B** Mean firing frequency of D1-MSNs was decreased by NN414, which was normalized by Glibenclamide (one-way ANOVA, F_(2, 32)_ = 4.65, *p* = 0.0169; Holm-Šídák’s multiple comparisons test, Ctr vs. NN414, *p* = 0.0425; NN414 vs. NN414+Gliben, *p* = 0.0425; Ctr vs. NN414+Gliben, *p* = 0.83). Datapoints in the bar graph are mean firing frequencies elicited by the 100-150 pA depolarizing steps. **C** Rheobase of D1-MSNs was not significantly affected by NN414, but decreased by Glibenclamide (Kruskal-Wallis test, K-W = 7.6, p = 0.0224; Dunn’s multiple comparisons test, Ctr vs. NN414, *p* = 0.422; NN414 vs. NN414+Gliben, *p* = 0.019; Ctr vs. NN414+Gliben, *p* = 0.63). **D** Same representation as in **A** for D2-MSNs. F–I curves showing that K-ATP channel activation with NN414 did not alter D2-MSN firing (two-way RM ANOVA; main treatment effect, *F*_(2, 21)_ = 0.183, *p* = 0.834); *n* = 11, 12, 9 cells from *N* = 2 male and *N* = 1 female mice. **E** Mean firing frequency of D2-MSNs was not altered by NN414, but increased by Glibenclamide (one-way ANOVA, F_(2, 21)_ = 3.87, *p* = 0.037; Holm-Šídák’s multiple comparisons test, Ctr vs. NN414, *p* = 0.105; NN414 vs. NN414+Gliben, *p* = 0.634; Ctr vs. NN414+Gliben, *p* = 0.053). Datapoints in the bar graph are mean firing frequencies elicited by the 100-150 pA depolarizing steps. **F** Rheobase of D2-MSNs was not significantly affected by NN414 or Glibenclamide (Kruskal-Wallis test, K-W = 1.77, *p* = 0.413). **G** Left, examples of voltage responses elicited in MSNs upon somatic current depolarizations (protocol at the bottom). D1-MSNs were patch-clamped with an intracellular solution containing 1.5 mM ATP in the presence of the K-ATP channel blocker glibenclamide (Gliben, 3 µM) and treated with vehicle (Ctr) or 100 nM rotenone (Rot). Right, F-I curves showing that K-ATP channel blockade prevented the effect of complex I inhibition on cell firing (two-way RM ANOVA; main treatment effect, *F*_(1, 21)_ = 1.182e-005, *p* = 0.9973); *n* = 11, 12, 9 cells from *N* = 3 male and *N* = 1 female mice. **H–I** In the presence of the K-ATP channel glibenclamide, rotenone treatment did not alter mean firing frequency (two-tailed unpaired *t* test, *t*_20_ = 1.63, *p* = 0.1191) or rheobase (two-tailed Mann-Whitney test, *U* = 55, *p* = 0.493). **J** Experimental scheme for assessment of coping behavior in the forced swim test in mice cannulated in the NAc for delivery of rotenone and glibenclamibe. **K** Animals were infused with vehicle (Veh), rotenone (Rot), glibenclamide (Gliben), and a combination of rotenone and glibenclamide (Rot+Gliben) prior to the exposure to the forced swim test. Passive coping behavior was evaluated as the %time spent immobile, i.e., without actively trying to escape the water bucket (two-way RM ANOVA for the last 4 min; main treatment effect, *F*_(3, 36)_ = 3.72, *p* = 0.02); *N* = 10, 11, 10, 9 male mice. **L** K-ATP blockade prevented the increase in passive coping behavior induced by complex I inhibition. Bar graph shows the comparison of mean values of %time spent immobile during the last 4 min of test (Kruskal-Wallis test, *K-W* = 8.35, *p* = 0.039; Dunn’s *post hoc* test, Veh vs. Rot, *p* = 0.014; Rot vs. Gliben, *p* = 0.036; Rot vs. Rot+Gliben, *p* = 0.019; *p* > 0.1 for the other comparisons). Data are represented as mean ± SEM, with datapoints corresponding to single cells/animals. Symbols indicate statistical difference between groups: #*p* < 0.1; *p < 0.05.

To test whether K-ATP channel activation modulates intrinsic excitability in the NAc, we used the SUR1/Kir6.2-preferring K-ATP channel opener NN414 [31]. In D1-MSNs recorded with a low-ATP intracellular solution, preincubation with NN414 (20 µM) significantly decreased firing frequency, consistent with a reduction in intrinsic excitability. This effect was reversed by co-application of the K-ATP channel blocker glibenclamide (20 µM), which restored excitability to control levels (Fig. 5A–C). In contrast, NN414 did not significantly affect the excitability of D2-MSNs (Fig. 5D–F).

Finally, to assess whether K-ATP channels contribute to the effects of mitochondrial inhibition on MSN excitability, we tested whether K-ATP channel blockade could prevent the changes induced by complex I inhibition. Preincubation of slices with glibenclamide fully prevented the effects of rotenone on intrinsic excitability (Fig. 5G–I).

These findings indicate that Kir6.2-containing K-ATP channels in MSNs act as key effectors for the reduction in excitability induced by mitochondrial complex I inhibition, and further suggest that these channels are more readily recruited in D1-MSNs. However, K-ATP channels are also present in cholinergic interneurons and in dopaminergic presynaptic terminals in the striatum [30, 32–34], both of which can influence dopamine release. This raises the possibility that alterations in MSN excitability following rotenone exposure could be indirectly mediated by mitochondrial inhibition in other NAc cell types with a consequent decrease in tonic dopamine levels—an effect that would also be prevented by broad K-ATP channels blockade with glibenclamide. To exclude this possibility (i.e., an indirect action of dopamine on MSN firing), we recorded from D1-MSNs in the presence of the D1 receptor antagonist SCH-23390 (10 µM). As expected, D1-MSN firing output was overall reduced, in particular due to apparent inactivation of Na^+^ currents in response to large depolarizations (Fig. S3A). However, firing frequency and rheobase were still significantly affected by rotenone (Fig. S3A-B).

We also confirmed that the rotenone effect was independent of tonic GABAergic or glutamatergic transmission, as it persisted in the presence of blockers for GABA_A_Rs, AMPARs, and NMDARs (Fig. S3C-D). Additionally, synaptic GABAergic (Fig. S3E-F) and glutamatergic (Fig. S3G-H) currents evoked by electrical stimulation in MSNs were unaffected by 100 nM rotenone in terms of peak amplitude and paired-pulse ratio, ruling out changes in presynaptic release. Overall, these results support the conclusion that the impact of complex I inhibition on MSN excitability is cell-autonomous and mediated by postsynaptic K-ATP channels in MSNs.

### K-ATP channel blockade prevents rotenone-induced passive coping behavior

We have previously shown that acute pharmacological manipulations of mitochondrial complexes in the NAc bidirectionally affect behavioral outcomes in a social dominance paradigm in rats [16]. Here, we investigated whether acute complex I inhibition impacts the exertion of effort in the forced swim test (FST), a paradigm used to assess active versus passive coping strategies under inescapable stress. Active coping in this context involves sustained motor output to maintain escape-directed behavior, which is energetically demanding and therefore relevant to our investigation of mitochondrial function. Consistent with this, our previous work has demonstrated that proper metabolic function in the NAc is required for the expression of active coping behavior in the FST [17, 35], supporting the relevance of this paradigm to our study. In male mice implanted with bilateral cannulas for drug delivery to the NAc, we infused rotenone or its vehicle prior to the exposure to a forced swim test (Fig. 5J). Passive coping was quantified as the time spent immobile, i.e., not attempting to escape. Rotenone administration significantly increased immobility, indicating enhanced passive coping. Importantly, this effect was reversed by co-administration of the K-ATP channel blocker glibenclamide in the NAc (Fig. 5K–L). *Post mortem* analyses indicated that rotenone infusion did not induce neurotoxicity (Fig. S4A–E). In addition, glibenclamide alone did not affect behavior in the forced swim test (Fig. 5F–G), or general locomotor activity, as assessed by the distance moved in a novel open field (Fig. S4F). These results support the view that an acute bioenergetic deficit engages K-ATP channels in NAc MSNs, thereby reducing their excitability and limiting their contribution to sustaining effortful behavior.

## Discussion

The present study identifies key mechanisms by which acute mitochondrial dysfunction alters the intrinsic excitability of NAc MSNs and, in turn, the engagement of NAc circuits in effortful behavior. Using the mitochondrial complex I inhibitor rotenone, we show that mitochondrial impairment reduces MSN intrinsic excitability in a cell-autonomous manner. This effect is mediated by K-ATP channels and is prevented by replenishing cytoplasmic ATP levels, consistent with rotenone’s established role in impairing oxidative phosphorylation and decreasing ATP production [36, 37].

Previous work examined rotenone’s effects on mesolimbic neurons mainly in the context of Parkinson’s disease models [33, 34, 38–40]. In one study, Bao and colleagues reported that submicromolar concentrations of rotenone acutely depolarized striatal MSNs, reducing input resistance, an effect attributed to transient receptor potential (TRP) cation channel activity [40]. Notably, while blocking K-ATP channels prevented input resistance reduction, it did not counteract rotenone-induced depolarization, leading to the conclusion that rotenone’s impact on MSNs were driven by elevated H₂O₂ rather than ATP depletion. By contrast, our findings reveal that rotenone reduces MSN excitability through ATP depletion and K-ATP channel activation, without affecting input resistance changes. These diverging results likely reflect methodological differences: Bao et al. applied rotenone after a baseline recording in cells patched with high intracellular ATP (3 mM), whereas we tailored conditions to model acute changes in mitochondrial function using unpaired comparisons of vehicle- and rotenone-treated cells, and using low intracellular ATP (1.5 mM) to avoid confounding effects of ATP replenishment due to prolonged cell dialysis. Consequently, we observed an impact of rotenone on MSN excitability that may have been masked in the prior study [40], a result corroborated by our own high-ATP experiments.

Our study also reveals cell-type-specific responses to mitochondrial impairment in NAc neurons. D1-MSNs exhibited a marked reduction in excitability following rotenone exposure, even at low concentrations, an effect that was mimicked by a K-ATP channel opener, while D2-MSNs were less affected. Possible explanations for this difference include variations in post-translational modification or trafficking of K-ATP channels, or distinct metabolic coupling and ATP/ADP dynamics between the two MSN subtypes. These possibilities highlight intriguing avenues for future research into subtype-specific regulation of excitability and the role of bioenergetic sensing in striatal circuits. Given the distinct contributions of MSN subtype in motivated and reward-driven behaviors [11, 41–44], this differential vulnerability to mitochondrial inhibition may help explain how bioenergetic alterations in the NAc contribute to motivational deficits seen in conditions such as depression. Additionally, we show that MSNs selectively express the Kir6.2 subunit, while Kir6.1 was absent from MSNs and instead localized to cholinergic interneurons [30]. This cell-type-specific distribution of K-ATP channel subunits may account for the relative resilience of cholinergic interneurons to rotenone-induced excitability changes, consistent with previous findings in glucose deprivation models [30], likely due to the lower sensitivity of Kir6.1 than Kir6.2 subunit to ADP/ATP fluctuations [45]. Because acetylcholine levels are low in acute slice preparations due to active cholinesterases, our recordings may underestimate the contribution of this neuromodulator to MSN function. While we found that rotenone does not alter the intrinsic excitability of putative cholinergic interneurons, this cell-autonomous measure does not capture potential effects on neurotransmitter release. Therefore, we cannot exclude that mitochondrial complex I inhibition affects acetylcholine release from cholinergic interneuron axon terminals, thereby indirectly modulating MSN activity in vivo. Similar limitations apply to other neuromodulatory inputs to the NAc, including dopamine and adenosine, whose release dynamics and extracellular availability are not fully preserved under our ex vivo conditions.

In vivo, we found that NAc rotenone infusion promoted passive coping behavior in a forced swim test, an indicator of effort-related deficits. This effect was prevented by co-administration of the K-ATP blocker glibenclamide, which did not affect baseline locomotion or passive coping on its own, suggesting that complex I inhibition within the NAc specifically affects coping behavior through K-ATP channel activation. These findings are consistent with studies linking mitochondrial function to social dominance and motivated behavior [16, 17, 19, 20], reinforcing the role of mitochondrial health in behavioral regulation through MSN excitability.

In summary, our results indicate K-ATP channels as rapid molecular sensors of mitochondrial function in MSNs, translating ATP deficits into decreased excitability and increased passive coping behavior. This mechanism establishes a direct link between cellular energy state and behavioral output, providing insight into how alterations in mitochondrial bioenergetics in the NAc may contribute to effort-related deficits observed in psychiatric conditions such as mood disorders.

Behavioral experiments were conducted in male mice only. Some reports indicate that stress-related behaviors in females can be modulated by estrous cycle stage [46, 47], and we considered that including females without explicit cycle monitoring could introduce additional biological variability. In light of the ethical and severity-related constraints of the forced swim test, we therefore limited the sample to male mice. For the ex vivo slice experiments, potential estrous cycle effects were controlled by comparing control and rotenone conditions in slices obtained from the same animal. However, we agree with the importance of this point and foresee that future studies should directly investigate whether mitochondrial modulation of MSN excitability and passive coping behaviors exhibits sex- or hormone-dependent differences.

## Supplementary information

Supplemental Information

## Data Availability

The datasets generated during and/or analysed during the current study are available from the corresponding author on reasonable request.
