# Supplementary material for: Energy-dependent modulation of nucleus accumbens output via K-ATP channel activity
Source: Neuropsychopharmacology. 2026 Apr 30;51(11):2238–47. doi: 10.1038/s41386-026-02429-8 (PMC13598099; doi:10.1038/s41386-026-02429-8)
Supplement: Supplementary file 1 [file 41386_2026_2429_MOESM1_ESM.pdf]

**Supplemental material for**

# Energy-dependent modulation of nucleus accumbens output via K-ATP channel activity

Simone Astori et al.

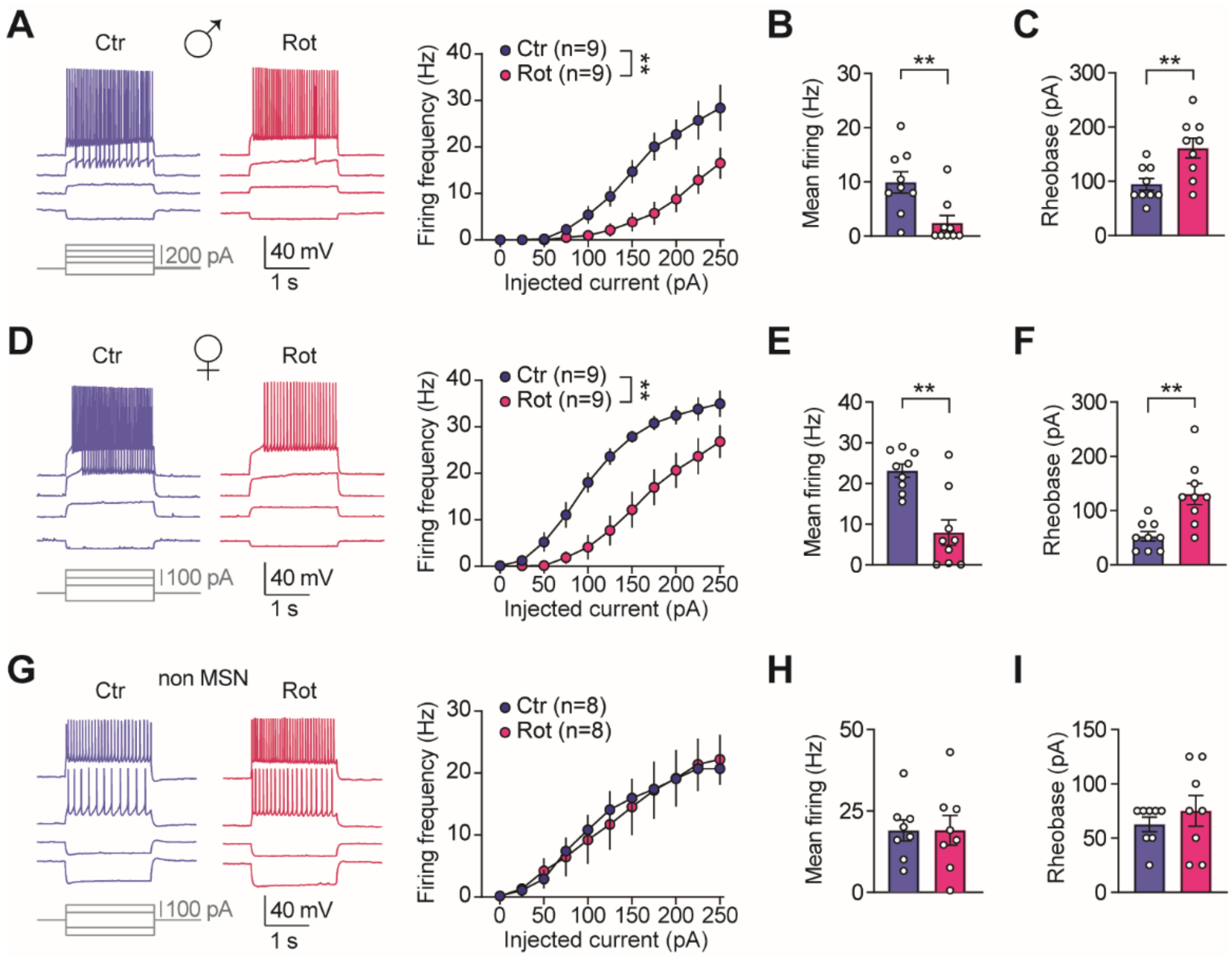


**Fig. S1: Low dose of rotenone affects MSN intrinsic excitability in both sexes, but does not impact NAc cholinergic interneurons.**

**A** Left, examples of voltage responses elicited in NAc MSNs from male mice upon somatic current depolarizations (protocol at the bottom). MSNs were preincubated with vehicle (Ctr) or 100 nM rotenone (Rot). Right, average firing frequency vs. current relationships (F-I curves) for Ctr and Rot cells, showing a significant reduction in firing of MSNs treated with rotenone (two-way RM ANOVA; main treatment effect, $F_{(1,\ 16)}$ = 9.74, $p$ = 0.0066); n = 9 cells from N = 4 male mice.

**B** Complex I inhibition reduced mean firing frequency of NAc MSNs from male mice (two-tailed Mann-Whitney test, $U$ = 10, $p$ = 0.0053), quantified as the average firing rate evoked by 100–150 pA depolarizing current injections.

**C** Complex I inhibition increased the rheobase of NAc MSNs from male mice (two-tailed unpaired $t$ test, $t_{16}$ = 3.145, $p$ = 0.0063).

**D** Left, examples of voltage responses elicited in NAc MSNs from female mice upon somatic current depolarizations (protocol at the bottom). MSNs were preincubated with vehicle (Ctr) or 100 nM rotenone (Rot). Right, average firing frequency vs. current relationships (F-I curves) for Ctr and Rot cells, showing a significant reduction in firing of MSNs treated with rotenone (two-way RM ANOVA; main treatment effect, $F_{(1,\ 16)}$ = 15.05, $p$ = 0.0013); n = 9 cells from N = 3 female mice.

**E** Complex I inhibition reduced mean firing frequency of NAc MSNs from female mice (two-tailed Mann-Whitney test, $U$ = 8, $p$ = 0.0027), quantified as the average firing rate evoked by 100–150 pA depolarizing current injections.

**F** Complex I inhibition increased the rheobase of NAc MSNs from female mice (two-tailed unpaired *t* test, $t_{16} = 3.645$, $p = 0.0022$).

**G** Left, examples of voltage responses elicited in putative NAc cholinergic interneurons upon somatic current depolarizations (protocol at the bottom). MSNs were preincubated with vehicle (Ctr) or 100 nM rotenone (Rot). Right, average firing frequency vs. current relationships (F-I curves) for Ctr and Rot cells, showing lack of rotenone effect on cell firing (two-way RM ANOVA; main treatment effect, $F_{(1, 13)} = 0.1799$, $p = 0.678$); n = 8, 8 cells from N = 6 male and N = 1 female mice.

**H-I** In putative cholinergic interneurons, rotenone treatment did not alter mean firing frequency (two-tailed unpaired *t* test, $t_{10.36} = 0.5$, $p = 0.627$), rheobase (two-tailed Mann-Whitney test, $U = 25$, $p = 0.736$).

Data are represented as mean ± SEM, with datapoints corresponding to single cells. Symbols indicate statistical difference between groups: $^{**}p < 0.01$.

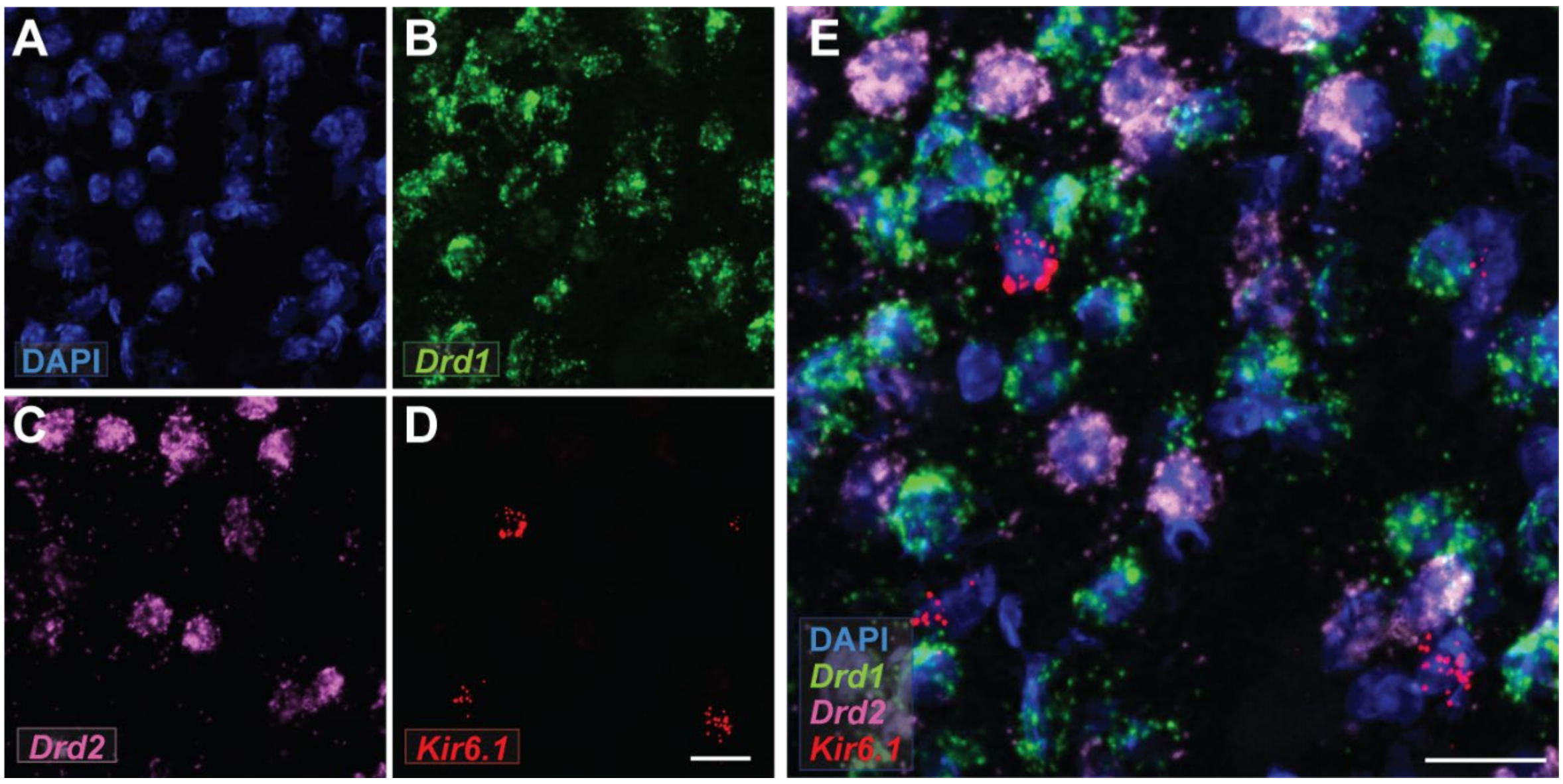


**Fig. S2: Kir6.1-containing K-ATP channels are expressed in a subpopulation of non-MSN cells.**

**A-E** Example images of DAPI staining and *in situ* mRNA hybridization for *Drd1*, *Drd2* and *Kir6.1* mRNA labeling in the NAc, with merged channels in **E**, showing that Kir6.1-containing K-ATP channels are expressed in a sparse subpopulation of cells in the NAc. Scale bars are 20 µm.

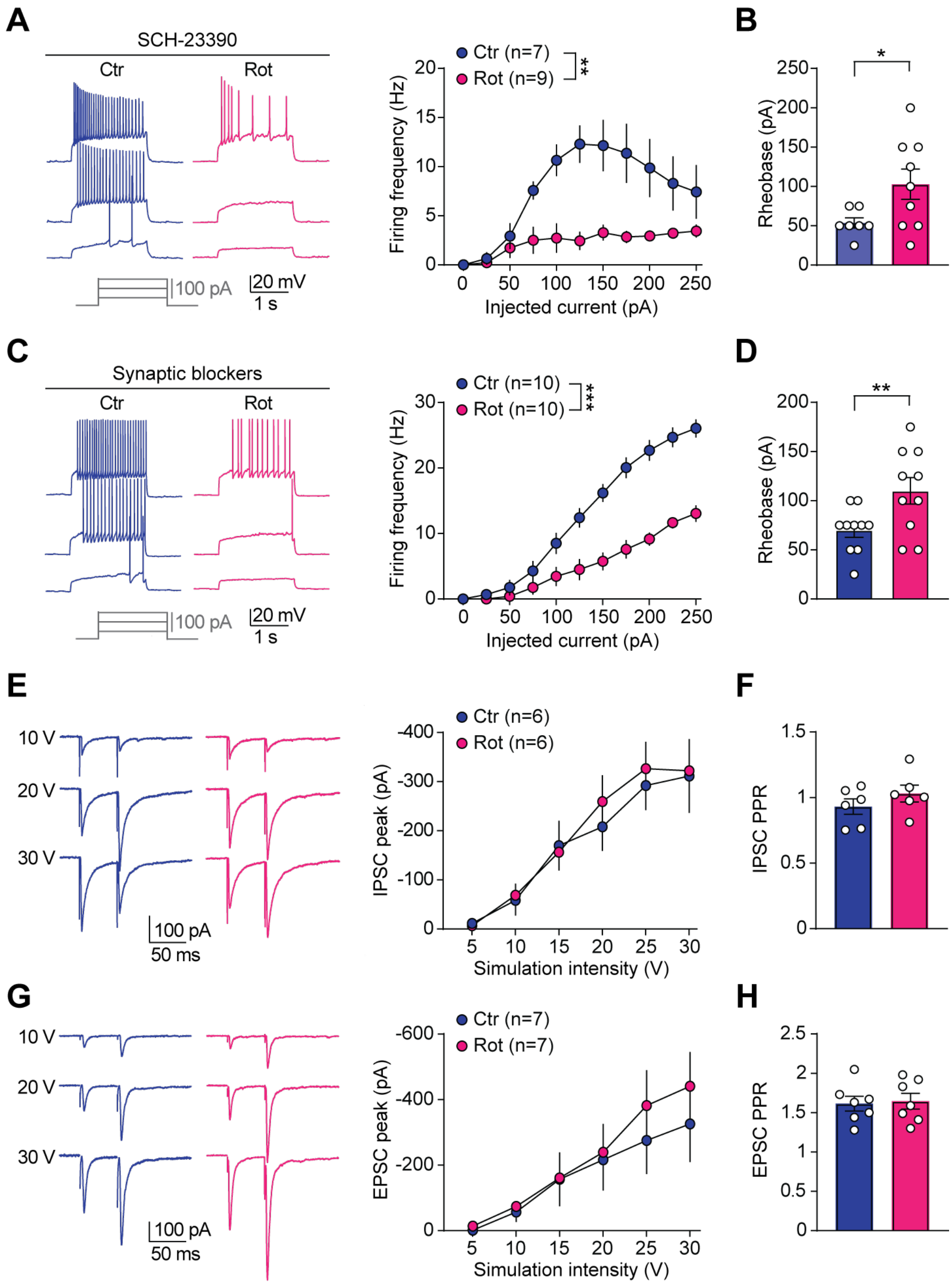


**Fig. S3: Mitochondrial I inhibition effect of MSN intrinsic excitability are cell autonomous.**

**A** Left, examples of voltage responses elicited in NAc D1-MSNs in the presence of the D1 receptor antagonists SCH-23390 (10 µM). D1-MSNs were preincubated with vehicle (Ctr) or 100 nM rotenone (Rot). Right, F-I curves for Ctr and Rot cells, showing a significant reduction in firing of D1-MSNs treated with 100 nM rotenone (two-way ANOVA; main treatment effect, $F_{(1, 14)} = 13.49$, $p = 0.0025$); n = 7, 9 cells from N = 2 male mice.

**B** Complex I inhibition increased the rheobase of D1-MSNs also in the presence of the D1 receptor antagonists SCH-23390 (two-tailed unpaired *t* test, $t_{9.76} = 2.42$, $p = 0.037$).

**C** Left, examples of voltage responses elicited in NAc D1-MSNs in the presence of the synaptic blockers (100 µM picrotoxin, 10 µM DNQX and 100 µM D,L-APV). D1-MSNs were preincubated with vehicle (Ctr) or 100 nM rotenone (Rot). Right, F-I curves for Ctr and Rot cells, showing a significant reduction in firing of D1-MSNs treated with 100 nM rotenone (two-way RM ANOVA; main treatment effect, $F_{(1, 18)} = 32.19$, $p < 0.0001$); n = 10, 10 cells from N = 3 male mice.

**D** Complex I inhibition increased the rheobase of D1-MSNs also in the presence of synaptic blockers (two-tailed unpaired *t* test, $t_{13.79} = 2.6$, $p = 0.021$).

**E** Left, examples traces depicting inhibitory postsynaptic currents (IPSCs) evoked in MSNs by electrical stimulation of increasing intensity. Right, input-output curves show lack of effect of rotenone treatment (100 nM) of IPSC peak amplitude (mixed-effects model; main treatment effect, $F_{(1, 10)} = 0.096$, $p = 0.762$); n = 7, 7 cells from N = 2 male and N = 1 female mice.

**F** Rotenone treatment did not affect paired-pulse ratio (PPR) of IPSCs (two-tailed unpaired *t* test, $t_{9.92} = 1.15$, $p = 0.27$).

**G** Left, examples traces depicting excitatory postsynaptic currents (EPSCs) evoked in MSNs by electrical stimulation of increasing intensity. Right, input-output curves show lack of effect of rotenone treatment (100 nM) of IPSC peak amplitude (mixed-effects model; main treatment effect, $F_{(1, 12)} = 0.203$, $p = 0.66$); n = 6, 6 cells from N = 2 male mice.

**H** Rotenone treatment did not affect paired-pulse ratio (PPR) of EPSCs (two-tailed unpaired *t* test, $t_{11.93} = 0.23$, $p = 0.82$).

Data are represented as mean ± SEM, with datapoints corresponding to single cells. Symbols indicate statistical difference between groups: *$p < 0.05$, **$p < 0.01$, ***$p < 0.001$.

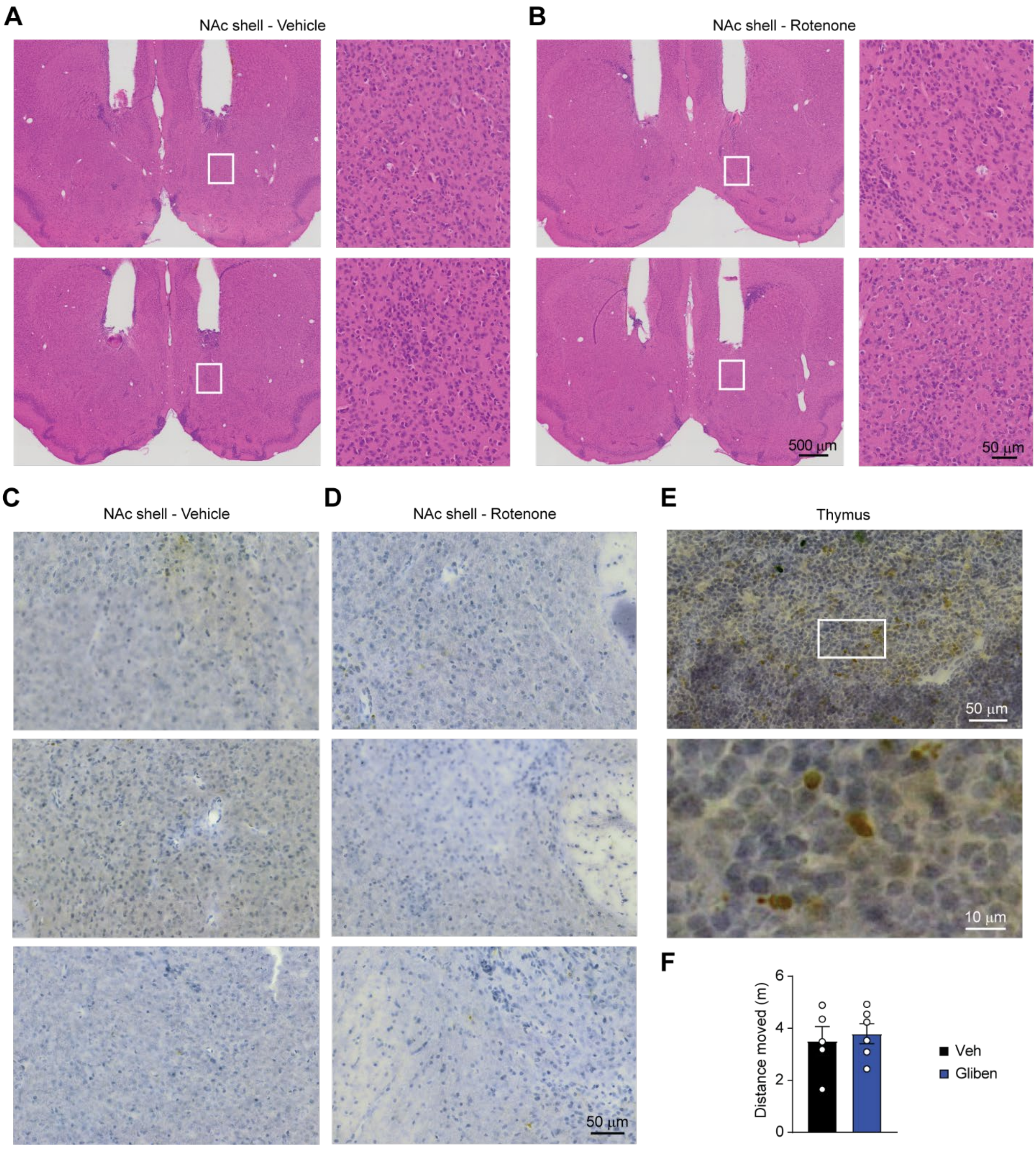


**Fig. S4: Lack of neurotoxic effects of rotenone infusion and of locomotor effects of glibenclamide.**

**A-B** Examples images from hematoxylin-eosin (HE) staining from mice infused with vehicle or rotenone, with high-magnification insets, indicating lack of evident cell damage in the infused areas.

**C-E** Examples images from cleaved caspase 3 staining in infused areas from the NAc and comparison with immunoreactive cells from the thymus.

**F** Glibenclamide infusion in the NAc did not alter global locomotor activity, as indicated by the comparable total distance moved in an open field by mice infused with vehicle (Veh) or with glibenclamide (Gliben)(two-tailed unpaired *t* test, $t_{7.39} = 0.42$, $p = 0.67$). Data are represented as mean ± SEM, with datapoints corresponding to single animals; N = 5 male mice.
